# Different Executive Functions Support Different Kinds of Cognitive Flexibility: Evidence From 2‐, 3‐, and 4‐Year‐Olds

**DOI:** 10.1111/cdev.12468

**Published:** 2015-12-11

**Authors:** Emma Blakey, Ingmar Visser, Daniel J. Carroll

**Affiliations:** ^1^ The University of Sheffield; ^2^ The University of Amsterdam

## Abstract

Improvements in cognitive flexibility during the preschool years have been linked to developments in both working memory and inhibitory control, though the precise contribution of each remains unclear. In the current study, one hundred and twenty 2‐, 3‐, and 4‐year‐olds completed two rule‐switching tasks. In one version, children switched rules in the presence of conflicting information, and in the other version, children switched rules in the presence of distracting information. Switching in the presence of conflict improved rapidly between the ages of 3 and 3.5 years, and was associated with better working memory. Conversely, switching in the presence of distraction developed significantly between the ages of 2 and 3 years, and was associated with better inhibitory control.

Cognitive flexibility describes the ability to adapt our thoughts and behavior in response to changes in our goals or our environment. It is commonly conceptualized as a complex, later developing ability that is made possible by improvements in inhibitory control and working memory (Chevalier et al., 2012; Cragg & Chevalier, 2009; Garon, Bryson, & Smith, 2008). However, while there is increasing evidence that there are important associations between cognitive flexibility and inhibitory control and working memory, the nature of these associations remains poorly specified.

Developmental studies have been highly informative about cognitive flexibility, particularly when focusing on the preschool years (e.g., Diamond, Carlson, & Beck, 2005; Muller, Dick, Gela, Overton, & Zelazo, 2006). During the preschool period children first demonstrate the ability to guide and to change their actions in line with explicit rules—a fundamental milestone in engaging in systematic goal‐directed behavior. The crucial enabling development of the preschool years has often been characterized as the emergence of the ability to overcome behavior based on an initial rule, in order to produce behavior guided by a new rule (e.g., Zelazo, 2006). For example, on a range of preschool cognitive flexibility measures, including the Dimensional Change Card Sort task (DCCS; Zelazo, 2006) and Shape School (Espy, 1997), 3‐year‐olds are able to sort colored shapes by a single rule (e.g., color) during the preswitch phase. However, when the rule changes in the postswitch phase (e.g., to sorting by shape), 3‐year‐olds are unable to reliably switch to the new rule. In contrast, 4‐year‐olds are able to sort correctly when the rule changes (Zelazo, 2006). This 3‐ to 4‐year shift—often characterized as a change from perseverative responding to flexible responding—has been presented as a crucial development in early cognition (Munakata, Snyder, & Chatham, 2012).

Explanatory accounts of the emergence of cognitive flexibility have tended to exclusively focus on either increases in inhibitory control or increases in working memory. All accounts have sought to explain why 3‐year‐olds perseverate on measures of cognitive flexibility and 4‐year‐olds do not. For example, the attentional inertia account (Diamond et al., 2005; Kirkham & Diamond, 2005) explains 3‐year‐olds' failure to switch rules as arising from poor inhibitory control. This account argues that following a rule change, 3‐year‐olds are unable to inhibit their attention to the no‐longer relevant dimension. In contrast, working memory accounts explain the same difficulty as arising from immature working memory. For example, the graded representations account explains 3‐year‐olds' failure to switch rules as arising from an inability to maintain the current rule in the face of competition from the previously relevant rule (Kharitonova & Munakata, 2011; Munakata, 2001). This account argues that because children fail to maintain the current rule strongly in their working memory, the previous, no‐longer relevant rule is selected instead as the basis for behavior. In a similar vein, goal‐neglect accounts also focus on working memory, arguing that young children make switching errors because they fail to maintain the goal of the current task (Chevalier & Blaye, 2008; Marcovitch, Boseovski, Knapp, & Kane, 2010; Towse, Lewis, & Knowles, 2007).

There are, therefore, two plausible but fundamentally different explanations offered to explain cognitive flexibility during the preschool period. Distinguishing between the two accounts is difficult, since both working memory and inhibitory control accounts make similar predictions for how 3‐ and 4‐year‐old children will perform on most current measures of cognitive flexibility. Nor is the focus on perseverative errors particularly helpful. Perseveration can be explained as children failing to either inhibit their attention to the previously relevant dimension or maintain in working memory the new task rule. Resolving the impasse between these different accounts is a primary concern for cognitive flexibility research, and indeed continues to be a question of great importance in the current literature (e.g., Chevalier et al., 2012; Dick, 2014; Ionescu, 2012). However, research using existing paradigms has been unable to make much progress in this regard. It is this issue that the present article addresses. We now set out three specific steps that will allow us to better understand the emergence of cognitive flexibility.

First, it is essential to be more precise about what is meant by “cognitive flexibility.” Within the preschool literature, cognitive flexibility is typically operationalized as the ability to sort a series of stimuli first by one rule, and then by another. However, alongside this basic demand, many paradigms also include the additional requirement for children to resolve within‐stimulus conflict. Response conflict arises when a stimulus possesses properties that prompt both possible rules on a given task, in other words, when it is possible to match a stimulus both by a new rule and by the initial sorting rule. In this situation, both perceptual aspects of the stimulus (e.g., color and shape) are relevant according to one of the two possible rules on the task (see Figure 1 for an illustration). Most cognitive flexibility tasks therefore—whether deliberately or inadvertently—make *two* demands of children: They must change their sorting behavior from using one rule to using another rule, and they must resolve the within‐stimulus conflict between the previous dimension and the new dimension. These tasks might inadvertently create the impression that resolving conflict is the only way to learn about the development of cognitive flexibility. However, even in the absence of response conflict, switching rules is far from a trivial demand for young children (Brooks, Hanauer, Padowska, & Rosman, 2003; Chevalier & Blaye, 2008). Keeping these distinct task demands separate is essential if we are to fully understand the developmental trajectory of cognitive flexibility. As such, in the current study we separate these two demands by examining children's ability to switch rules in the presence of (a) *conflicting* information and (b) *distracting* information.

**Figure 1:**
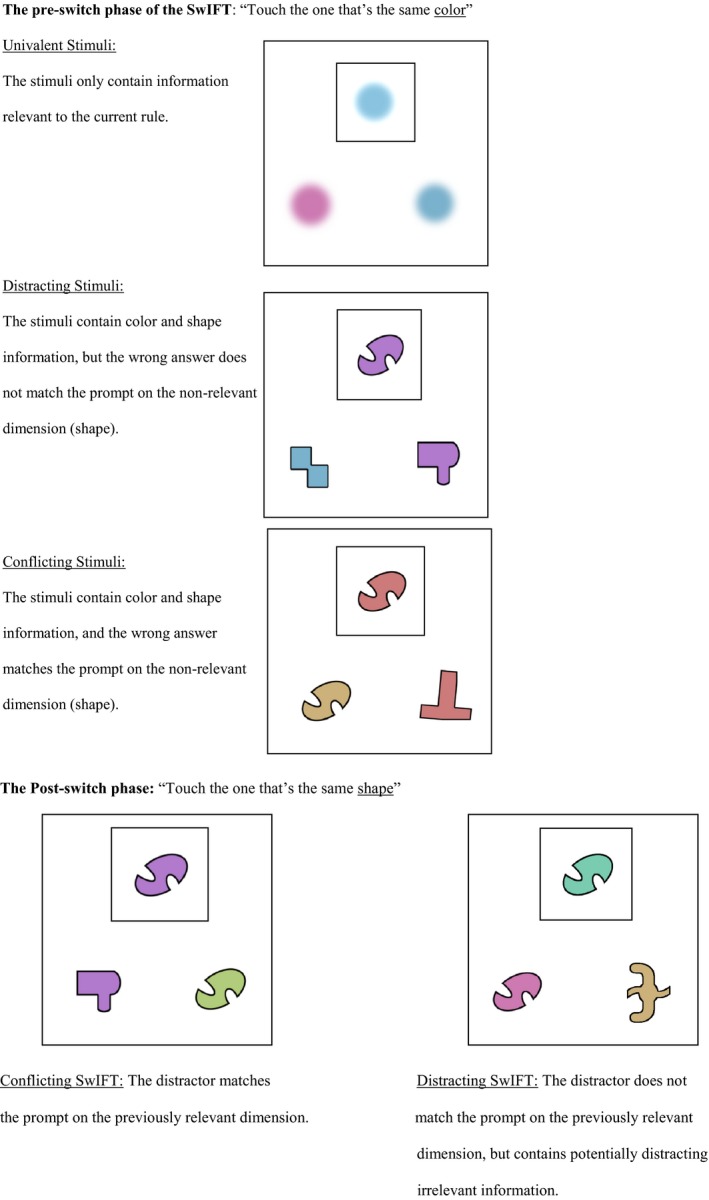
The preswitch and post‐switch phase of the Switching, Inhibition, and Flexibility task (SwIFT).

A further advantage of this dichotomy is that it allows us to distinguish between two distinct kinds of cognitive flexibility errors that would otherwise be conflated: perseverative errors, where children inappropriately persist with a previous rule, and distraction errors, where children fail to maintain their current sorting rule. Although standard measures of cognitive flexibility do not allow these types of error to be measured independently, there is clear evidence to suggest that this distinction is a crucial one. For example, Chevalier and Blaye (2008) assessed switching in preschoolers using the PAST‐3, a paradigm requiring an intradimensional switch (e.g., from sorting blue pictures to yellow pictures). The task allowed perseverative errors and distraction errors to be distinguished. They found that in 3‐year‐olds, distraction errors were as common as perseverative errors. This remains a promising yet underused approach to further understanding cognitive flexibility.

Second, it is important to study development prior to the 3‐ to 4‐year period. Examining switching in 2‐year‐olds is likely to be particularly informative, since the relative paucity of research with this age group means that little is known about the early emergence of cognitive flexibility. Two‐year‐olds have been tested on very simple measures of inhibitory control (Carlson, Mandell, & Williams, 2004) and working memory (Hughes & Ensor, 2007). However, most cognitive flexibility paradigms are too complex to use with children younger than 3 years. A paradigm with reduced incidental demands that would allow the study of cognitive flexibility from an earlier age would be particularly useful.

Third, looking at individual differences in inhibitory control and working memory will allow us to better understand their role in the development of cognitive flexibility. While previous attempts to use this approach have been limited in number, they have nevertheless been extremely promising. For example, Marcovitch et al. (2010) examined how preschoolers' performance on a battery of working memory tasks predicted their ability to switch rules. They found that 4‐year‐olds with better working memory were better able to switch rules than children with poorer working memory. Chevalier et al. (2012) examined the separate contributions of both working memory and inhibitory control to local costs and mixing costs on the Shape School. Three‐ to 5‐year‐olds completed a mixed block of trials, requiring them to make multiple switches between two rules. Children with better working memory and inhibitory control had lower mixing costs (i.e., the cost of repeating a rule in a *mixed* block, rather than a pure block). There was no effect of working memory or inhibitory control on local costs (i.e., the cost of switching rules between trials). To our knowledge, these are the only studies to have taken an individual differences approach to examine the development of cognitive flexibility, so a further extension of this approach is likely to be informative.

The current study combined all three of these approaches to examine how individual differences in working memory and inhibitory control relate to different kinds of cognitive flexibility in 2‐ to 4‐year‐olds. Children were tested on separate measures of working memory and inhibitory control, as well as on two variants of a cognitive flexibility task. To examine cognitive flexibility, we used the Switching, Inhibition, and Flexibility task, or SwIFT (FitzGibbon, Cragg, & Carroll, 2014). This is a simple rule‐switching task in which children must decide which of two colorful shapes matches a prompt image on the relevant dimension for that trial (either color or shape, with the rule changing halfway through the task). The SwIFT is administered on a touch‐screen computer, so that responses are simple for even very young children. To ensure that the task was appropriate for 2‐year‐old children, it included a staggered preswitch phase where stimuli gradually increased in complexity: from simple univalent stimuli, to distracting stimuli, to conflicting stimuli (see Figure 1). The SwIFT has simple verbal demands (using the prompt “Touch the one that's the same [color/shape]”). In addition, in order to be able to identify subtle developmental changes, our age‐related analyses used 6‐month age bands (cf. Gerstadt, Hong, & Diamond, 1994) to examine 2.5‐, 3‐, 3.5‐, and 4‐year‐olds.

In order to study both the ability to switch rules in the presence of conflicting information and the ability to switch rules in the presence of distracting information, two different variants of the SwIFT task were used, differing only in the stimuli used. We systematically varied the type of stimuli children sorted in the postswitch phase to examine the development of switching (a) in the presence of response conflict (the Conflicting SwIFT) and (b) in the presence of distracting information (the Distracting SwIFT; shown in Figure 1). In the Conflicting SwIFT, children had to switch rules while sorting stimuli with response conflict. In the postswitch phase of this task—as in tasks such as the Shape School or DCCS—it was possible for children to continue to sort by the previous, no‐longer relevant dimension (in other words, it was possible for them to perseverate). In contrast, in the Distracting SwIFT, children had to switch rules while sorting stimuli with distracting, task‐irrelevant information. In this version, children must still update their sorting behavior. However, they cannot continue to match by the previously relevant dimension, since no postswitch stimuli match on that dimension. In other words, it was not possible for children to perseverate. Any errors children make in the postswitch phase of this condition reflect distraction errors. In addition, while it is often informative to categorize children based on their performance because there are often clearly homogenous groups (such as switchers and perseverators), it is important to confirm such potentially arbitrary categories using statistical techniques. Therefore, in the current study, latent Markov models were fitted to the data; this allowed us to discriminate between different types of errors that vary as a function of task and children's age (Visser, 2011).

## Method

### Participants

One hundred and twenty children took part in the study (58 males and 62 females). Data from a further seven children were excluded: One child was later diagnosed with specific language impairment, three failed to understand the task instructions, and three did not complete a full set of test trials. The remaining sample was split into four age groups: twenty‐six 2.5‐year‐olds (*M*_age_ = 2 years 8 months, range = 2 years 4 months to 2 years 11 months), forty‐two 3‐year‐olds (*M*_age_ = 3 years 3 months, range = 3 years to 3 years 6 months), twenty‐one 3.5‐year‐olds (*M*_age_ = 3 years 9 months, range = 3 years 7 months to 3 years 11 months), and thirty‐one 4‐year‐olds (*M*_age_ = 4 years 3 months, range = 4 years to 4 years 6 months). Children were recruited either from a database of local families who had expressed an interest in participating in research or from local nursery schools. All children were monolingual, were from homes or schools in working‐class and middle‐class areas of the United Kingdom, and were predominantly White British. Participating families received a small gift as a token of appreciation for taking part. Informed consent was obtained from parents before the testing began. Ethical approval was obtained from the department's ethics subcommittee. Testing took place between January 2013 and March 2014.

### Procedure

Children were tested in a single session, either in the University Developmental Lab with their caregiver present, or in a quiet area of their nursery. Children first played a short warm‐up game, which also served to make sure that they understood the words *color*,* shape*, and *same*. They then completed four tasks in a fixed order: one variant of the SwIFT, the inhibitory control task, the working memory task, and finally the second variant of the SwIFT. To minimize the chance of data loss on the most developmentally appropriate switching task, the version of the SwIFT that children completed first differed according to age: 2.5‐ and 3‐year‐olds completed the Distracting SwIFT first, whereas 3.5‐ and 4‐year‐olds completed the Conflicting SwIFT first. In addition, since there is no single age‐appropriate inhibitory control task that can be used from 2 to 4 years of age, younger and older children completed different measures of inhibitory control.

### Assessing Cognitive Flexibility

#### The SwIFT

This was a rule‐switching task presented on a touch screen computer, in which children had to decide which of two colorful shapes matched a prompt image on the relevant dimension for that trial (either color or shape). The task was presented on an Iiyama ProLite touch screen connected to a standard PC running E‐Prime Software (PST, Pittsburgh, PA). The task first began with 3 practice trials and then, the preswitch phase of 12 trials using one matching rule, and finally a postswitch phase of 8 trials using a different matching rule.

Each trial began with a prompt stimulus appearing at the top of the screen. After a delay of 1000 ms, two response stimuli appeared in the lower corners of the screen. One stimulus was the target (the correct response, as it matched the prompt on the currently relevant dimension), and the other was a distractor (the incorrect response). The distractor and target were equally likely to appear on the left or right. Children were prompted to respond with the recorded instruction “Touch the one that's the same (color/shape).” Children responded by touching their chosen image. When children selected the correct response, a musical cartoon animation appeared in place of the stimulus selected. When children selected the incorrect response, the display disappeared, no animation was played, and the next trial began. If the child did not make a response, the experimenter repeated the prompt. Rule order was fully counterbalanced. Two different versions of the SwIFT were used, differing only in the type of stimuli children sorted by in the postswitch phase.

#### Conflicting SwIFT

In this variant of the SwIFT, the postswitch stimuli had response conflict: The incorrect response option matched the prompt image on the no‐longer relevant dimension (and would thus appear to be the correct response if children failed to select the appropriate sorting rule).

#### Distracting SwIFT

In this variant of the SwIFT, the postswitch stimuli did not have response conflict, since the incorrect response option did not match the prompt image on the no‐longer relevant dimension. Instead, the distractor stimuli contained distracting, task‐irrelevant shape or color information.

### Assessing Inhibitory Control

#### Reverse Categorization Task

The 2.5‐ and 3‐year‐olds completed the Reverse Categorization task, a measure of inhibitory control appropriate for younger preschoolers (Carlson et al., 2004). The task used 2 boxes (1 yellow and 1 blue) and 12 cubes (6 yellow and 6 blue). In the introductory phase, children were told to place cubes into the box of the same color. In the testing phase, children were told to place the cubes into the box of the other color. A rule reminder was provided on every trial. The dependent variable was the number of cubes correctly sorted in the testing phase of the task.

#### Day–Night Stroop Task

The 3.5‐ and 4‐year‐olds completed the Day–Night Stroop task, a measure of inhibitory control in older preschoolers (Simpson & Riggs, 2005). The task used a pair of picture cards, one depicting a book and the other depicting a car. The experimenter explained that when she said *book*, children should point to the car picture, and when she said *car*, they should point to the book picture. Children then completed four practice trials, with feedback. The testing phase had 12 trials, without feedback, where *book* and *car* were said aloud in a fixed order with no more than three consecutive repetitions of one of the words. The dependent variable was the number of correct responses.

### Assessing Working Memory

#### Spin the Pots Task

All children completed the Spin the Pots task (Hughes & Ensor, 2007). Eight visually distinct pots with lids were arranged on a rotating tray. Children watched the experimenter put colorful stickers in six of the pots, and the two empty pots were pointed out before children began searching. Each search trial began with the experimenter covering the tray with a cloth and then rotating the tray for a few seconds. If the children found a sticker in the pot they selected, they kept it. After each search attempt, the tray was again covered and rotated, and a new search trial began. The task ended either once children had found all six stickers, or after 16 trials. The dependent variable was the total number of trials taken.

## Results

### Preliminary Analyses

A series of one‐way analyses of variance (ANOVAs) found no effect of gender on working memory, on inhibitory control, or on cognitive flexibility (all *p*s > .05). A two‐way ANOVA found no effect of rule order on accuracy on either SwIFT variant, and no interaction (all *p*s* *> .05). One‐sample *t* tests conducted separately for each age group found that all age groups sorted at above‐chance levels in the preswitch phase of the SwIFT (all *p*s < .001), and thus could perform well on the basic task. To look first at how SwIFT accuracy varied by age, one‐way ANOVAs were conducted to examine the effect of age group on overall accuracy on each SwIFT task. There was no effect of age group on Conflicting SwIFT accuracy, *F*(3, 111) = 1.93, *p *>* *.10 (see below for why this may be the case). There was a significant effect of age group on Distracting SwIFT accuracy, *F*(3, 114) = 13.65, *p *<* *.001. Bonferroni post hoc tests found that this was due to improvements in the ability to switch in the presence of distracting information between the ages of 2.5 years (*M *=* *5.31, *SD *= 1.64) and 3 years (*M *=* *6.36, *SD *= 1.59), *p *=* *.02.

### Identifying SwIFT Performance Types Using Latent Markov Models

Prior research with preschool switching tasks has identified large individual differences that are categorical in nature (e.g., Dauvier, Chevalier, & Blaye, 2012; van Bers, Visser, van Schijndel, Mandell, & Raijmakers, 2011). For example, children may respond either by switching correctly, by perseverating with the initial rule, or by fluctuating between the two rules. It is difficult to measure these differences using techniques such as ANOVA that depend on measuring central tendency. To illustrate, if half the children in a sample perseverated, and half switched successfully, the overall sample mean of four out of eight might appear to indicate that the entire sample was performing at chance. The systematic, within‐group difference in performance would be lost. Hence, in order to better identify individual differences in children's postswitch performance, we used latent Markov models (Visser, 2011). We used latent Markov models to analyze the trial‐by‐trial sequences of children's performance during the postswitch phase using depmixS4 (Visser & Speekenbrink, 2010), an add‐on package for the statistical analysis software R (R Core Team, 2014). We follow a similar approach to van Bers et al. (2011) and van Bers, Visser, and Raijmakers (2014) to identify discrete categories of performance type during the postswitch phase (what we refer to in the model as “strategies”). The word *strategy* is used here solely in the sense that children perform consistently across a number of trials (see Rickard, 2004). In addition, latent Markov models can also identify any transitions between these strategies over the course of the postswitch phase.

To differentiate between the different types of errors that children made in the Conflicting SwIFT and the Distracting SwIFT, latent Markov models were used to model trial‐by‐trial accuracy in the postswitch phase of each task. Latent Markov models with 1–4 states were fitted on the Conflicting and Distracting SwIFT data separately. The first step was to determine the optimal number of states of the latent Markov models. Models with a larger number of states are better at capturing the data (as evidenced by a higher log‐likelihood), but at the cost of adding more parameters. Model selection statistics such as Akaike's information criterion (AIC; Akaike, 1973) and the Bayesian information criterion (BIC; Schwarz, 1978) are used to find the right balance between better capturing the data while retaining a parsimonious model. Here, we use the small sample corrected version of the BIC (Nylund, Asparouhov, & Muthén, 2007; Sclove, 1987). As our main interest was to determine whether different participants used different initial strategies, we also tested models in which the initial probability of starting in one particular strategy depended on the age of the participants.

### Model‐Based Analyses: Conflicting SwIFT

The modeling analyses revealed that for the Conflicting SwIFT, the “3‐state + age” model had the best (i.e., lowest) BIC value and hence we proceed with interpretation of that model here. Table 1 shows the goodness‐of‐fit statistics of the fitted latent Markov models with 1–4 states.

**Table 1:** Goodness‐of‐Fit Statistics for the Latent Markov Models of the Conflicting Switching Inhibition and Flexibility Task

Model	LL	npar	AIC	BIC	BICw
1 state	−619.57	1	1,241.15	1,242.80	0.00
Regression (age)	−608.44	2	1,220.80	1,224.19	0.00
2‐state	−517.68	5	1,045.36	1,053.60	0.00
2‐state + age	−515.55	6	1,043.10	1,052.99	0.00
3‐state	−508.17	9	1,034.34	1,049.18	0.00
3‐state + age	−500.85	9	1,019.70	1,034.54	0.94
4‐state	−505.51	10	1,031.02	1,047.51	0.00
4‐state + age	−494.62	14	1,017.24	1,040.32	0.05

Note

The abbreviation “npar” denotes the number of parameters; it is corrected for parameters estimated at their boundary values (0 or 1). The small‐sample version of the BIC is reported here. Finally, BICw denotes the BIC weights. AIC = Akaike's information criterion; BIC = Bayesian information criterion.

There are three noteworthy findings in the parameter estimates given in Table 2. First, the three states of the model have clearly identifiable strategies. The first state has a postswitch probability correct of around 0.52, approximately chance level. We refer to this as the “mixed responding” state. The second state has a probability correct of 0.07, that is, an almost complete absence of switching. We refer to this as the “perseveration” state. Finally, the third state has a probability correct of 0.93, corresponding to almost completely accurate responding. We refer to this as the “switch” state. Note that on tasks such as the DCCS, typically only two types of performance are recognized (perseveration and switching).

**Table 2:** Parameter Estimates of Each Model in the Conflicting Switching Inhibition and Flexibility Task Demonstrating the Transition Matrix Values (i.e., the Probability of Transitioning From One Strategy to Another During the Postswitch Phase)

Model	Switchers	Mixed responders	Perseverators
Switchers (0.93)	1.0	0.00	0.00
Mixed responders (0.52)	0.00	0.98	0.03
Perseverators (0.07)	0.09	0.00	0.91

Note

Initial state probabilities of switching are given in parentheses.

Second, the probability of remaining in the switch state, once entered, is 1.0. In other words, once participants adopt this optimal strategy, they do not revert to other strategies after that—there is no *un*learning once the correct strategy has been adopted. Furthermore, the only positive transitions are (a) from mixed responding to perseveration, and (b) from perseveration to switch. This suggests that participants who start in the mixed responding state transition to the perseveration state *first*, and only then to the switch state. Crucially, there is no *direct* transition from the mixed responding state to the switch state. Perseveration therefore appears to represent an intermediate stage in the development of successful switching.

**Figure 2:**
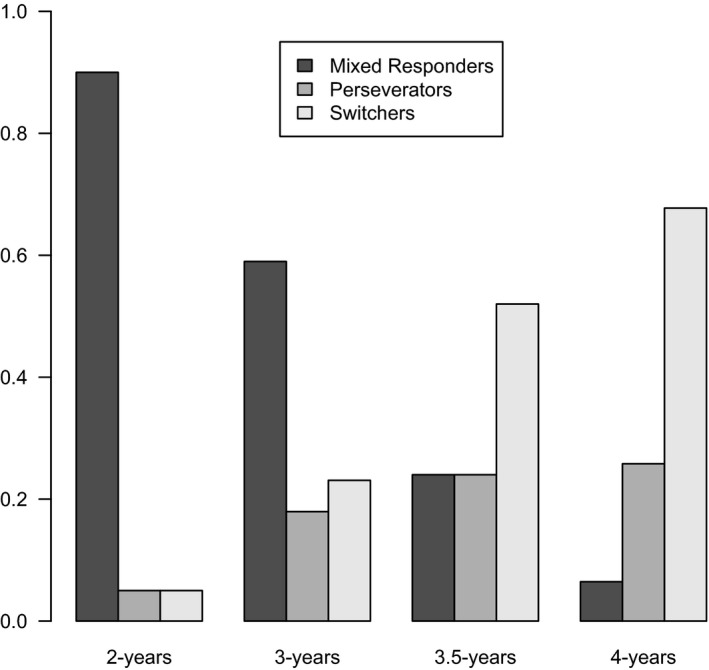
The proportion of children starting in each strategy on the Conflicting Switching, Inhibition, and Flexibility task as a function of age group.

Third, the initial probabilities model parameters are informative about the developmental pathway suggested by these data. Figure 2 displays the proportion of participants starting in each strategy as a function of age group. Figure 2 was created by first assigning participants to strategies for each observation using posterior state sequences (Visser, 2011), and then computing the proportion of assigned strategies within age groups. An interesting developmental pattern is revealed: Most 2‐year‐olds show a mixed responding strategy, which decreases with age. The perseveration strategy slowly increases with age, albeit in a relatively small group of children. Finally, the proportion of children initially using the correct switch strategy clearly increases with age.

To test the hypothesis that working memory predicts which strategy children use on the Conflicting SwIFT, two different models were compared. In one, age was included as a covariate on children's initial strategy; in the other, working memory was used as a covariate. The BIC values were 1,002.81 and 995.46 for the “age” model and the working memory model, respectively. These values indicate that the “working memory” model provides a better fit to the data, and thus that working memory predicts initial strategy better than age. (Note that these BIC values are not comparable to the ones in Table 2 because for these models five children were dropped due to missing working memory data.) Model‐based analyses with inhibitory control as a covariate were not computed because children completed a different inhibitory control task depending on their age.

### Model‐Based Analyses: Distracting SwIFT

**Figure 3:**
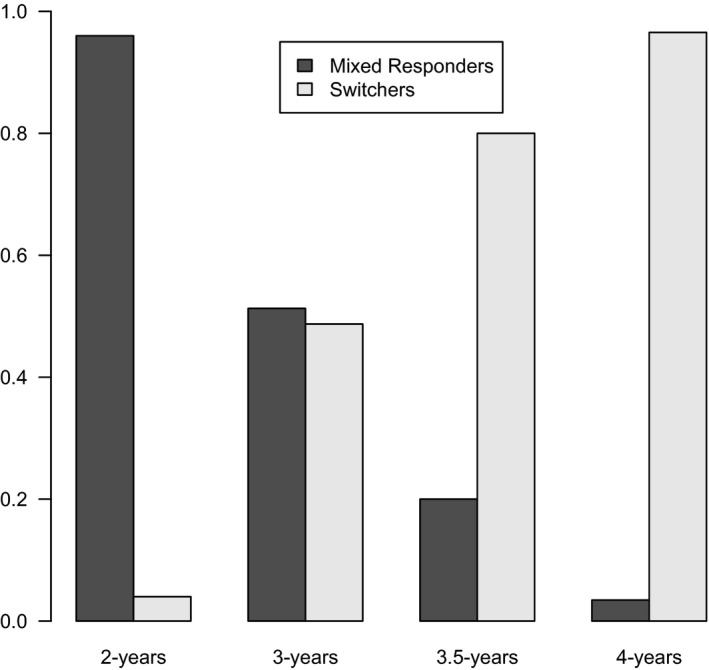
The proportion of children starting in each strategy on the Distracting Switching, Inhibition, and Flexibility task as a function of age group.

The modeling approach for the Distracting SwIFT is analogous to that for the Conflicting SwIFT. Table 3 shows the goodness‐of‐fit statistics of the fitted latent Markov models with one to three states. The “2‐state + age” model has the best (i.e., lowest) BIC value, and hence we proceed with interpretation of that model here. The parameter estimates are given in the Table 4. The two states of the model have clearly identifiable strategies. The first state has a probability correct on postswitch trials of around 0.63, corresponding to a “mixed responding” state. The second state has a probability correct of 0.96, corresponding to a “switch state.” The fact that the two‐state model is optimal confirms that children show two different patterns of responding on this task (switching and mixed responding). Figure 3 displays the proportion of children starting in each strategy as a function of age group: The mixed responding strategy gradually reduces between 2 and 4 years of age.

**Table 3:** Goodness‐of‐Fit Statistics for the Latent Markov Models of the Distracting Switching Inhibition and Flexibility Task

Model	LL	npar	AIC	BIC	BICw
1 state	−448.13	1	898.27	899.94	0.00
Regression (age)	−415.56	2	835.13	838.48	0.01
2‐state	−421.80	4	851.61	858.30	0.00
2‐state + age	−403.71	6	819.42	829.46	0.96
3‐state	−419.92	5	849.84	858.21	0.00
3‐state + age	−398.53	11	819.05	837.47	0.02

Note

The abbreviation “npar” denotes the number of parameters; it is corrected for parameters estimated at their boundary values (0 or 1). The small‐sample version of the BIC is reported here. Finally, BICw denotes the BIC weights. AIC = Akaike's information criterion; BIC = Bayesian information criterion.

**Table 4:** Parameter Estimates of Each Model in the Distracting Switching Inhibition and Flexibility Task Demonstrating the Transition Matrix Values

Model	Switchers	Mixed responders
Switchers (0.96)	1.00	0.00
Mixed responders (0.63)	0.05	0.96

Note

Initial state probabilities of switching are given in parentheses.

To test whether working memory predicted which strategy children used on the Distracting SwIFT, two models were compared: one in which age was included as a covariate on initial strategy, and one in which working memory was a covariate. The BIC values were 788.45 and 810.90 for the age model and the working memory model, respectively. These values indicate that the age model provides a better fit to the data, and hence that age better predicts initial strategy on the Distracting SwIFT than working memory.

### Individual Differences Analyses

Adopting the categories of switching performance established by the modeling analyses, children were grouped on the basis of their SwIFT performance into one of three categories: “switchers” (children who sorted 6–8 correct trials of 8), “mixed responders” (3–5 correct trials), or “perseverators” (0–2 correct trials). These categories were used to further examine relations with working memory and inhibitory control.

#### Contributions of Working Memory to Cognitive Flexibility

The modeling analyses indicated that some numerically higher scores on the Conflicting SwIFT (mixed responding, i.e., a score of around 4 of 8) reflected poorer cognitive flexibility than perseveration (i.e., a score of 0). Correlations were thus inappropriate for analyzing Conflicting SwIFT data. Therefore, these analyses examine how children's performance type on the Conflicting SwIFT (perseveration, mixed responding, switching) and age predict working memory performance on the Spin the Pots task.

A two‐way ANOVA was conducted to investigate whether age group and performance type on the SwIFT predicted children's performance on the Spin the Pots task. (Children were categorized as perseverators if they scored between 0 and 2 on the postswitch phase [*n = *27], as mixed responders if they scored between 3 and 5 [*n *=* *34], and as switchers if they scored between 6 and 8 [*n *=* *54]). With regard to the ability to switch rules in the presence of response conflict, there was a significant main effect of age, *F*(3, 100) = 2.92, *p *=* *.038, η^2^ = .06, and a significant main effect of Conflicting SwIFT performance type on Spin the Pots score, *F*(2, 100) = 5.23, *p *=* *.007, η^2^ = .08. There was no interaction between age and performance type, *F*(6, 100) = 0.90, *p *=* *.50. Bonferroni post hoc tests were used to follow up the significant main effect of SwIFT performance type. These showed the effect was driven by mixed responders (*M *=* *9.36, *SD* = 2.63) performing worse on the Spin the Pots task than switchers (*M *=* *7.31, *SD* = 1.39), with lower scores denoting better performance (*p *=* *.010). No other comparisons were significant (*p *>* *.10).

With regard to the ability to switch in the presence of distracting information, both performance‐type analyses and correlational analyses were conducted (as on the Distracting SwIFT, higher scores reliably correspond to better performance). Children were categorized as either switchers (*n *=* *31) or mixed responders (*n *=* *85). Two children categorized as perseverating were excluded, since it is not possible to match stimuli by the preswitch rule on this task, and their performance likely indicates a failure to understand the task. A two‐way ANOVA with age and performance type as factors found a significant effect of age on Spin the Pots score, *F*(3, 103) = 3.17, *p *=* *.03, η^2^ = .17. There was no significant effect of Distracting SwIFT performance type on Spin the Pots performance, *F*(2, 103) = .73, *p *=* *.48, and no interaction, *F*(4, 103) = 1.48, *p *=* *.21. In addition, Pearson's correlations found no correlation between switching in the presence of distraction and working memory in 2‐year‐olds, *r*(23) = .08, *p *>* *.10; 3‐year‐olds, *r*(40) = −.19, *p *>* *.10; 3.5‐year‐olds, *r*(21) = −.04, *p *>* *.10; or 4‐year‐olds, *r*(29) = −.27, *p *>* *.10.

#### Contributions of Inhibitory Control to Cognitive Flexibility

To investigate the relation between cognitive flexibility and inhibitory control, a series of one‐way ANOVAs were conducted for each age group (because children completed a different measure of inhibitory control depending on their age). With regard to switching in the presence of conflict, a one‐way ANOVA was conducted to look at whether performance type on the Conflicting SwIFT predicted Reverse Categorization scores in 2‐year‐olds. There was no effect of performance type on 2‐year‐olds' inhibitory control, *F*(2, 17) = 0.08, *p *>* *.10. As 3‐year‐olds performed near ceiling on the Reverse Categorization task, it was not possible to look at the relation between their inhibitory control and cognitive flexibility. For 3.5‐year‐olds and 4‐year‐olds, a two‐way ANOVA with age and performance type on the Conflicting SwIFT was run on Stroop accuracy. There was no effect of age, *F*(1, 43) = 0.01, *p *>* *.10, or of performance type, *F*(2, 43) = 0.84, *p *>* *.10.

With regard to switching rules in the presence of distracting information, similar ANOVAs were run as above. Pearson's correlations were also conducted for each age group. For 2‐year‐olds, there was a borderline‐significant effect of Distracting SwIFT performance type on Reverse Categorization scores, *F*(1, 22) = 4.03, *p = *.057, η^2^ = .15. Two‐year‐olds categorized as mixed responders (*n *=* *13; *M *=* *4.15, *SD *= 4.28) performed worse on the Reverse Categorization task than those categorized as switchers (*n *=* *11; *M *=* *8.18, *SD *= 5.55). In addition, there was a positive correlation between accuracy on the Distracting SwIFT and Reverse Categorization performance, *r*(25) = .43, *p *=* *.03. As 87% of 3.5‐year‐olds and 97% of 4‐year‐olds were able to switch on the Distracting SwIFT meaning there was little variance within performance types in the older children, only correlations were run between Stroop accuracy and total accuracy on the Distracting SwIFT. For 3.5‐year‐olds there was a positive correlation between accuracy on the Distracting SwIFT and Stroop accuracy, *r*(19) = .48, *p *=* *.038. For 4‐year‐olds, there was no correlation between accuracy on the Distracting SwIFT and Stroop accuracy, *r*(30) = .10, *p *>* *.10.

In summary, inhibitory control was related to the ability to switch in the presence of distracting information for both 2.5‐ and 3.5‐year‐olds. Conversely, children who showed mixed responding when switching in the presence of response conflict had poorer working memory than children who were able to switch rules successfully.

## Discussion

The aim of this study was to investigate the emergence of cognitive flexibility during early childhood by combining an individual differences approach with a wider, younger age range, and using finer grained definitions of cognitive flexibility. Three findings in particular shed new light on this topic. First, when asked to switch rules, 2.5‐ and 3‐year‐olds' postswitch performance was most typically characterized by mixed responding (i.e., not sorting consistently by either available sorting rule), rather than by perseveration. Second, the different kinds of cognitive flexibility showed distinct developmental trajectories. Switching in the presence of response conflict (as measured by the Conflicting SwIFT) showed an increase in perseveration and a decrease in mixed performance during the preschool period. In contrast, switching in the presence of distracting information (as measured by the Distracting SwIFT) improved significantly between the ages of 2.5 and 3 years. Third, inhibitory control and working memory were both associated with cognitive flexibility, but importantly, each was associated with a distinct kind of cognitive flexibility. Taken together, these results substantially advance our understanding by identifying distinct developmental trajectories of two kinds of cognitive flexibility, and furthermore, by offering evidence that these trajectories are underpinned separately by inhibitory control and working memory. We now consider these findings in further detail.

For 2.5‐ and 3‐year‐olds, sorting during the preswitch phase was simple. Their chief difficulty on both kinds of switching task was in maintaining systematic rule‐governed behavior when the sorting rule changed—as evidenced by their mixed responding both in the Distracting SwIFT and Conflicting SwIFT. Mixed responding in the presence of response conflict was common in 2.5‐ and 3‐year‐olds, but decreased substantially in 3.5‐year‐olds. The 2.5‐ and 3‐year‐olds also frequently showed mixed responding when switching in the presence of distracting information. This improved significantly between the ages of 2 and 3 years. This shows that significant developments in cognitive flexibility occur at around 3 years of age, highlighting the importance of examining cognitive flexibility development in younger children. The model‐based analyses confirmed that for the Conflicting SwIFT, children show one of three performance types (switching, mixed responding, or perseverating), whereas for the Distracting SwIFT, children can be categorized in one of two ways (switching or mixed responding). This is noteworthy, since comparable model‐based analyses examining 3‐ to 5‐year‐olds' performance on the DCCS found only two performance types (switching or perseverating) with a transitional group of children that shift from perseveration to switching (van Bers et al., 2011). The model‐based analyses in the present study justify the classification of children as “mixed” performers and indicate that perseveration is likely to be an intermediate state between mixed performing and successful switching.

The low incidence of perseveration in the present study stands in contrast to some previous research that reports high rates of perseveration (e.g., Zelazo et al., 2003). While the lack of perseveration when switching in the presence of distraction is unsurprising—since it is not possible to continue to sort by the previously relevant rule—it is noteworthy that there was so little perseveration from children switching in the presence of response conflict. Under these conditions, perseverative errors are perfectly possible (and according to some accounts of cognitive flexibility, to be expected). However, rates of perseveration were generally quite low: 30% of 2.5‐year‐olds, 17% of 3‐year‐olds, 38% of 3.5‐year‐olds, and 19% of 4‐year‐olds. This is a finding of particular interest, since it includes data from an age group younger than the 3‐ to 4‐year range typically studied. Results from the modeling analyses showed that perseveration was confined to only a small group of children, and interestingly, that this type of error gradually increased between the ages of 2 and 4 years (in contrast to mixed responding, which decreased with age). We note that the SwIFT and the DCCS both share the requirement that children categorize stimuli first by one rule, then by another. However, the SwIFT has reduced incidental demands. This may indicate that the low incidence of perseveration in the current study is because perseverative errors arise partly as a function of task demands incidental to switching.

Errors that are not perseverative in nature have received little attention in the literature, in part because many developmental paradigms are unable to detect them. Nevertheless, these types of errors have been documented in preschoolers, both on variants of the DCCS (Brooks et al., 2003; Chevalier & Blaye, 2008; Fisher, 2011; van Bers et al., 2011) and on other measures of preschool executive function (e.g., Dauvier et al., 2012; Towse et al., 2007). For example, Chevalier and Blaye (2008) found that when 3‐year‐olds could make either perseverative or distraction errors on a cognitive flexibility task, they were equally likely to make each kind of error. Our results offer further support to the idea that the ability to maintain the relevant task rule is a crucial demand on cognitive flexibility tasks—and to the suggestion that the importance of perseverative errors has been overestimated in prior work (Chevalier & Blaye, 2008).

Although mixed responding was a common pattern of performance on both the Conflicting SwIFT and Distracting SwIFT, it is likely that this pattern of performance arises for different reasons on each task. On the Conflicting SwIFT, when children were faced with competition from the previous rule, mixed responding was related to children's working memory. In addition, the modeling analyses revealed that working memory was a better predictor of performance type than age on the Conflicting SwIFT. This is a surprising finding, though it is consistent with previous research suggesting a link between working memory and switching in older children (Marcovitch et al., 2010), and reduced mixing costs in young children (Chevalier et al., 2012). The present results suggest a specific link between working memory and mixed responding errors. Interestingly, this supports previous research by Dauvier et al. (2012) who found that 5‐ and 6‐year‐olds' mixed responding on an alternating‐runs version of the DCCS was related to performance on a separate measure of working memory. Together, these results demonstrate that this pattern of performance is qualitatively different from perseveration. They highlight too the importance of working memory for not only updating rules (as in the alternating‐runs version of the DCCS) but also for maintaining systematic rule‐governed behavior (as on the SwIFT). The relation between working memory and mixed responding errors may be because children do not maintain the preswitch rule very strongly (as they do not perseverate), nor do they adopt the postswitch rule (as they do not sort correctly). The present data are consistent with working memory and goal‐neglect accounts of cognitive flexibility development that are based on children's performance on tasks with response conflict. These accounts posit that working memory supports cognitive flexibility by allowing children to maintain new task rules and use these to guide their behavior—particularly under conditions where there is conflict between previously relevant task rules and new task rules (Blackwell, Cepeda, & Munakata, 2009; Marcovitch et al., 2010).

On the Distracting SwIFT, children cannot continue to match by the preswitch rule, and so their errors must reflect distractibility rather than perseveration. This is an important observation: Even when the previously relevant dimension is entirely removed, it is still challenging for preschool children to switch to a new rule—and their attempts to switch can be disrupted by their difficulty in inhibiting distracting information. The cost incurred by nontask‐relevant information has been documented in only two previous studies we are aware of (Brooks et al., 2003; Chevalier & Blaye, 2008). Broader explanations of the emergence of cognitive flexibility have tended either to ignore this cost or to conflate it with the ability to resolve within‐stimulus conflict. The present study demonstrates clearly that these costs are separable, and should be treated as such in future research.

The results also allow us to refine our view of how inhibitory control supports cognitive flexibility. We found no association between inhibitory control and performance on the Conflicting SwIFT. Importantly, this stands in contrast to previous suggestions about the role of inhibitory control in cognitive flexibility. Specifically, the attentional inertia account suggests that young children perform poorly on switching tasks because they fail to inhibit attention to the no‐longer relevant dimension when presented with response conflict (e.g., Diamond et al., 2005). However, our results indicate that inhibitory control serves a different function in early cognitive flexibility. It appears to play a role in helping children to suppress distraction from task‐irrelevant information when sorting by a new rule. Specifically, 2.5‐ and 3.5‐year‐olds with stronger inhibitory control were better able to switch on the Distracting SwIFT than children with weaker inhibitory control. The results are consistent with previous research that reports inhibitory control to be related to aspects of switching that do not involve inhibiting attention to the previously relevant rule. Chevalier et al. (2012) found that 3‐ to 5‐year‐old children's inhibitory control was not related to switching (measured using local costs), but was related to their mixing costs on a switching task. The authors argue that inhibitory control may help children to resist distraction from nonrelevant features present in the stimuli. The current study shows that this observation extends to even younger children, and offers support for the idea that inhibitory control helps children to ignore task‐irrelevant information when they are required to switch or update task sets.

Two age‐related observations about inhibitory control must be addressed. First, inhibitory control in 4‐year‐olds was found to be unrelated to performance on the Distracting SwIFT. We suggest that this lack of association arises because 4‐year‐olds do not need to deploy inhibitory control in order to suppress their attention to distracting information, as by the age of 4, this demand is trivially easy for them. In support of this view, 97% of 4‐year‐olds could sort correctly in the presence of distracting information, suggesting that 4‐year‐olds are well able to switch rules while ignoring distractions. Second, 3‐year‐olds performed at near‐ceiling levels on the Reverse Categorization task, meaning we cannot make any definitive claims about inhibitory control at this age. It seems parsimonious and plausible to hypothesize that 3‐year‐olds would show a broadly comparable pattern of performance to the 2.5‐ and 3.5‐year‐olds. Unfortunately, to our knowledge there is no single age‐appropriate inhibitory control task that can be used from 2 to 4 years of age. The Reverse Categorization task was chosen because it has been used successfully with children aged 39 months, the mean age of our 3‐year‐olds group (e.g., Carlson et al., 2004). We also note that an alternative inhibitory task, the Day–Night Stroop task has high attrition rates when used with younger 3‐year‐olds (Simpson & Riggs, 2005). For that reason, the task would be unlikely to provide sufficient variance for an individual differences approach. Developing a measure of inhibitory control that can be used across a younger and broader age range remains a priority for future research.

When seeking to constrain theoretical accounts of the emergence of cognitive flexibility, it is important to note that working memory was *not* associated with the ability to switch rules in the presence of distracting information. In the absence of this observation, a rudimentary task analysis might suggest that holding in mind a strong representation of the current rule would be sufficient to allow children to ignore distractions efficiently. However, data from the present study do not support this view. Instead, they suggest that working memory is not integral to switching in the presence of distraction—likely because the lack of response conflict means there is no competition between the preswitch rule and the new postswitch rule to resolve. This helps to refine theoretical accounts that posit a role for working memory in cognitive flexibility development. Specifically, the results show that working memory is integral to switching only when there is a response conflict to resolve.

The present study offers a finer‐grained description of cognitive flexibility development, and shows how both working memory and inhibitory control contribute uniquely and distinctly to the emergence of cognitive flexibility in 2‐ to 4‐year‐olds. First, the present findings suggest that the role of perseveration has been overestimated in previous work and that it is not best seen as the starting point in the development of cognitive flexibility. Rather, before they perseverate, young children tend to respond with unsystematic mixed responding. Perseveration appears to be an error that increases over the preschool years, as mixed responding decreases. Second, the present results go beyond theoretical accounts positing that cognitive flexibility arises either from working memory or inhibitory control, to show that the specific contributions of working memory and inhibitory control vary as a function of differing task demands. Working memory is crucial for flexible behavior in the presence of response conflict. When faced with such response conflict in the present study, younger children tended to show mixed performance rather than perseveration, consistent with a failure to maintain the current task rule in working memory when updating behavior. These results also show that preschool children can be distracted by task‐irrelevant information when switching rules, even when response conflict is entirely absent. These difficulties were specifically related to individual differences in inhibitory control. Rather than inhibitory control helping children to suppress their attention to the previously relevant dimension, the current study suggests that it helps children to filter out task‐irrelevant information when switching. Together, these two distinct processes develop and combine to allow the emergence of complex and flexible rule‐driven behavior.
